# The Impact of Hybrid Therapy on Langerhans Cell Histiocytosis of the Mandible in an Older Male: A Case Report and Literature Review

**DOI:** 10.1155/crot/2996605

**Published:** 2025-10-22

**Authors:** Yoshifumi Matsumoto, Maki Akamatsu, Shinichi Ohba, Fumihiko Matsumoto

**Affiliations:** Department of Otorhinolaryngology, Juntendo University Faculty of Medicine, Tokyo, Japan

**Keywords:** case report, langerhans cells, mandible, steroid, surgery

## Abstract

**Background:**

The unusual disorder known as Langerhans cell histiocytosis (LCH), which is most frequently observed in children and young adults, is caused by the clonal proliferation of Langerhans cells. This disease is classified into several types depending on the extent of the lesion. Because it is rare diseases, there is no established standard treatment of this disease. In this report, we describe an extremely rare case of LCH that developed in the mandible of an older male. This is the first report in the world of a favorable outcome following surgical resection and local steroids administration.

**Case Report:**

The patient was a 75-year-old male who complained of swelling and pain in his mandible; however, there were no abnormal findings upon intraoral examination. Panoramic radiograph, computed tomography (CT), and magnetic resonance imaging (MRI) revealed osteolytic tumors. A diagnosis of LCH was confirmed based on the pathological findings of a cluster of Langerhans cells in a biopsy specimen of a submucosal tumor. Because the patient was elderly and the primary tumor was in the mandible, radiation therapy, chemotherapy, and systemic steroid administration were difficult to tolerate due to side effects. We performed combined treatment with surgical resection and local steroids injection. He was discharged from the hospital 1 week after the operation; the intraoral wound healed after 2 months, and the pain improved.

**Conclusions:**

This report suggests that a combination of surgical resection and local steroids administration is effective in treating LCH of the mandible in elderly patients.

## 1. Introduction

The characteristic feature of Langerhans cell histiocytosis (LCH) is the clonal proliferation of Langerhans cells. Abnormal Langerhans cells may infiltrate various tissues and organs. This proliferation is often accompanied by inflammatory infiltration, lymphocytes, and eosinophils [1]. LCH is classified in the category of Langerhans cells and other dendritic cell tumors [1, 2]. Nezelof et al. reported that histiocytosis X lesions are caused by the proliferation and dispersion of abnormal histiocytic Langerhans cells, identifying Birbeck granules as a marker; the name was then changed to LCH [1, 3]. The cause of LCH is unknown but may be infectious antigenic stimulation, genetic abnormalities, abnormal regulation of immunological responses, cytokine-mediated Langerhans cell proliferation, or clonal origin [1, 2]. Granulocyte macrophage colony-stimulating factor, interleukin-3, and tumor necrosis factor-α have all been implicated in the potential development of LCH; more recently, cytogenetic studies have shown the involvement of tumor suppressor genes (*p53*), oncogenes (*c-myc, H-ras*), cell surface immunological markers, and apoptosis factors [4].

It is known that LCH occurs at a rate of 0.5–5.4 cases per million people per year [1]. The incidence rate in adults is approximately 1–2 cases per million people [4], and it is more common in young individuals [5]. According to a large-scale cohort study of LCH, the onset of the disease in older adults is rare, with the median age at diagnosis being 36 years. The incidence of LCH in adults is reported to be approximately 10% of that observed in pediatric populations [6]. In particular, it was reported that the onset of single-system single-site (SS)-type LCH in older people is extremely rare [7].

We report a case of SS-type LCH that developed in the mandible of an older male, which is extremely rare; he was successfully treated with surgical resection and a combination of surgical resection and local steroid therapy. This study follows the Surgical Case Report (SCARE) criteria [8].

## 2. Case Presentation

**Figure 1:**
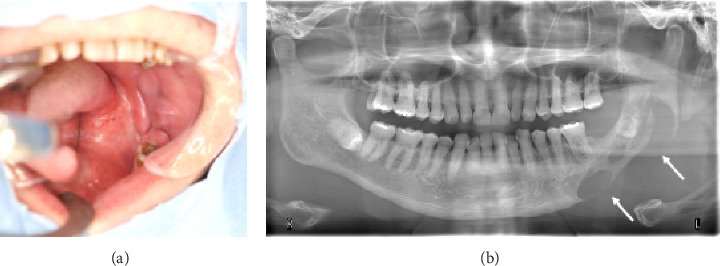
Oral findings at the time of examination. No abnormalities were observed in the mucous membrane (a). Panoramic radiograph showing destruction of the mandible. The white arrow indicates destruction of the mandible caused by the tumor (b).

A 75-year-old male was admitted to our hospital with a suspected malignant tumor of the mandible. The patient complained of swelling and mild pain in the lower jaw for the past 2 months. He had no history of systemic illness or allergies. Clinical examination revealed a firm, bony swelling affecting the left body of the mandible, slight tenderness, a smooth surface, and ill-defined borders. No cutaneous swelling was observed (Figure 1(a)). A mass was palpated on the external surface of the mandible, prompting a panoramic radiograph to assess for bone destruction, with suspicion of a malignant tumor.

**Figure 2:**
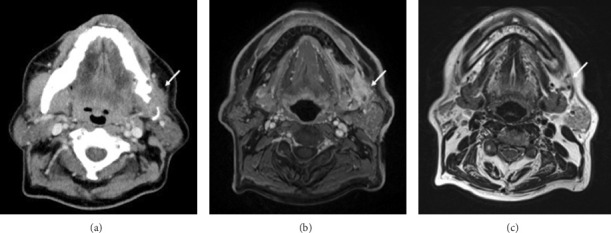
(a) The computed tomography (CT) images. (b, c) The magnetic resonance imaging (MRI) images. The lesions were highlighted by white arrows. CT scan showed a tumor involving destruction of the mandible (a). T1 weighted MRI demonstrated that the tumor with a high signal and irregular margins was observed (b). On T2 weighted MRI, the tumor was low signal, and there was also some signal change in the bone marrow (c).

Panoramic radiograph revealed an ill-defined radiolucent lesion in the left body of the mandible with discrete borders and no evidence of sclerosis; the associated teeth appeared normal (Figure 1(b)). Computed tomography (CT) demonstrated a soft tissue mass in the body of the mandible and the mandibular angle, accompanied by osteolytic changes. In some areas, there was also evidence of sclerosis of the mandible. The mandibular canal structure was disrupted. The findings suggested a malignant tumor originating in the mandible (Figure 2(a)). On magnetic resonance imaging (MRI), as with CT, tumor of the lower left gingiva was observed; the findings suggested a malignant tumor originating in the lower gingiva accompanied by periosteal and medullary invasion (Figures 2(b) and 2(c)).

**Figure 3:**
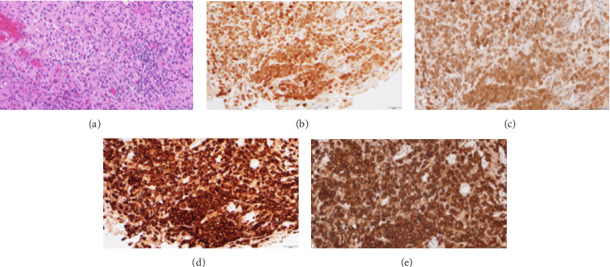
Pathological findings of biopsy specimens. Hematoxylin eosin staining showed clusters of Langerhans cells (× 400) (a). (b, c) Photomicrograph showing diffuse nuclear and cytoplasmic immunopositivity in > 95% of neoplastic cells, diffusely with moderate staining intensity, S-100 (× 200) (b) and (× 400) (c). (d, e) Photomicrograph showing diffuse cytoplasmic immunopositivity in > 95% of neoplastic cells, diffusely with strong staining intensity, CD1a, × 200 (d) and × 400 (e).

Core needle biopsy was performed under local anesthesia, and histopathological examination revealed Langerhans cells with a moderate amount of homogeneous, eosinophilic, granular cytoplasm, and distinct cell margins on hematoxylin and eosin staining (Figure 3(a)). In immunohistochemical staining, the specimen was positive for S-100 (Figures 3(b) and 3(c)) and CD1a (Figures 3(d) and 3(e)), confirming LCH. A systemic evaluation was conducted to exclude multifocal involvement by contrast-enhanced whole-body CT, which obtained negative results; the diagnosis was therefore concluded to be SS-type LCH.

The patient was initially kept under observation because he had only mild pain (Numerical Rating Scale [NRS] 1), but it later worsened (NRS 9); because of this, consequently, treatment was initiated within 2 months of the consultation.

**Figure 4:**
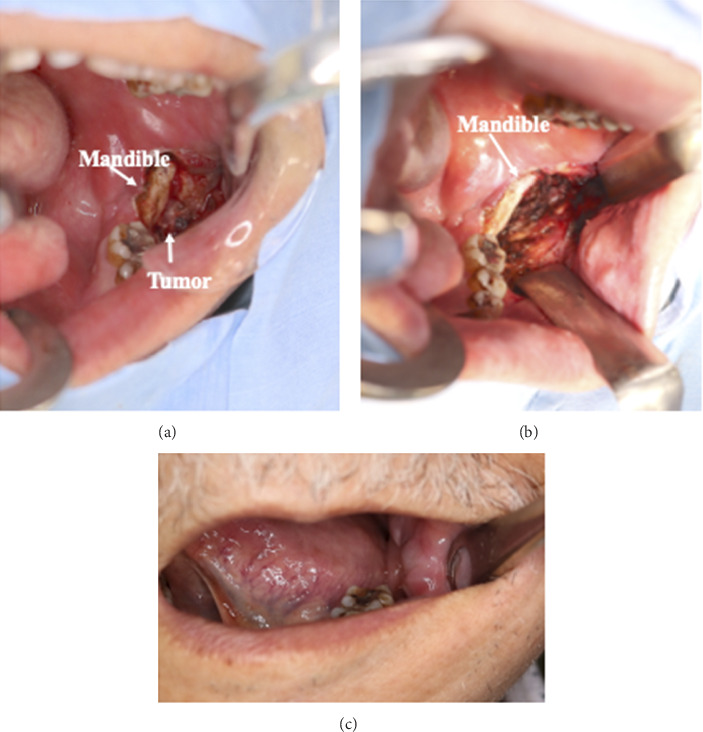
Surgical findings. A tumor was found on the lateral side of the mandible and the mandibular muscle process, through a mucosal incision in the posterior part of the mandible (a). Picture after resection. As much of the tumor was removed as was possible while preserving the bone (b). A photo of the wound 2 months after surgery. The patient has mild problems with opening the mouth and swelling, but the wound has already started to heal (c).

For LCH therapy, radiation therapy, chemotherapy (methotrexate and so on), surgical resection, and steroids administration (local or systemic administration) have been reported, depending on the extent of disease progression. In this case, the patient had LCH of the mandible, so there was a possibility of late effects such as osteonecrosis occurring with radiotherapy. Furthermore, as the patient was elderly, systemic chemotherapy and steroids administration were difficult to administer due to side effects such as immunosuppression. Thus, we selected surgical curettage and local steroids administration. Because the mandible had become thin due to osteolytic changes caused by LCH, we thought that the risk of fracture was high. Therefore, we limited the surgical curettage of the lesion without manipulating the bone. The procedure involved lesion curettage followed by direct steroid injection into the affected area. Surgery was performed under general anesthesia. The mucosa in the posterior part of the mandible was incised, the mandible and tumor were exposed, and a lesion was identified outside the mandible (Figure 4(a)). Given the high risk of fracture due to osteolytic changes, tumor excision was performed cautiously, as much of the tumor was removed as was possible while taking care not to damage the bone (Figure 4(b)). After excision, a local injection of dexamethasone (DEX) (6 mg) was administered around the wound, and the surgery was completed with mucosal closure using sutures. The patient had an uneventful postoperative recovery, and he was able to start eating orally the next day. He was discharged from the hospital on the 7th day after surgery. Steroids (DEX 6 mg) injections were administered weekly as an outpatient treatment, and complete epithelialization of the wound was observed 2 months after surgery along with improvement in pain (NRS 1). At the 10-month follow-up, clinical examination and CT imaging showed good wound healing (Figure 4(c)).

No written consent has been obtained from the patients as there is no patient identifiable data included in this case report/series.

## 3. Discussion

According to the 5th edition of the WHO classification, it is categorized as a tumor of the macrophage-dendritic cell lineage and is recognized as an inflammatory myeloid neoplasm driven by mutations in the mitogen-activated protein kinase (MAPK) pathway [2]. Clinically, LCH is classified into three types, single-system SS, in which a single organ is affected by a single lesion; single-system multisite (SM), in which a single organ is affected by multiple lesions, and multisystem multisite (MM), in which multiple organs are affected by multiple lesions. In the SS- and SM-types, the most common organs affected are the skull, femur, and pelvis; in the MM-type, skin and bone lesions are often combined, though the lungs, liver, lymph nodes, and central nervous system may be affected [4]. Oral LCH accounts for approximately 30% of all cases and often occurs in the mandible [9]. Hartman et al. reported that LCH involved the oral cavity in 114 (10%) of 1120 cases, with most of these cases in the mandibular molar region [5]. The clinical symptoms of LCH of the jawbone include bone pain and swelling, difficulty in opening the mouth, tooth mobility, fractures, and bone deformation. Panoramic radiograph findings show solitary or multiple punched-out bone lesions or bone destruction and often involve periosteal reactions [10]. In addition to panoramic radiography, both CT and MRI revealed osteolytic changes. The differential diagnoses included osteomyelitis, odontogenic tumor, and primary malignant tumor of the mandible. However, given the presence of not only osteolytic changes but also an associated soft tissue mass, a neoplastic lesion was primarily suspected, and a biopsy was performed. Based on the pathological diagnosis, systemic imaging studies were subsequently conducted, leading to the final diagnosis of SS-type LCH of the mandible in an elderly male.

The histopathological features of LCH include a diffuse or focal proliferation of Langerhans cells with characteristic kidney-shaped nuclei and eosinophilic cytoplasm, accompanied by variable infiltration of eosinophils. On immunohistochemistry, LCH exhibits positivity for S-100 protein and CD1a, and when examined under an electron microscope, it is characterized by the presence of Birbeck granules in the cytoplasm. Langerhans cell-derived tumors include LCH and Langerhans cell sarcoma. The latter is characterized by marked cellular atypia, pleomorphism, and nuclear division, with minimal eosinophilic infiltration; however, it is difficult to completely differentiate between them [9]. The MAPK pathway is an important part of the pathogenesis of LCH. This pathway, and in particular mutations in the BRAF (V600E) and MAP2K1 genes, is the most common genetic changes identified in LCH. These mutations lead to constitutive activation of the MAPK pathway, promoting cell proliferation and survival. Mutations in the MAPK pathway are identified in 80% of LCH cases and BRAF V600E in 50%–60% [10]. Other histiocytic diseases include juvenile xanthogranuloma (JXG), Erdheim–Chester disease (ECD), and Rosai–Dorfman disease (RDD). JXG is characterized by activating mutations in the MAPK pathway, such as MAP2K1, NRAS, KRAS, CSF1R, BRAF, and NTRK1 fusions. ECD is characterized by mutations in multiple MAPK pathway genes, such as BRAF V600E, ARAF, NRAS, KRAS, MAP2K1, and PIK3CA, and BRAF V600E mutations occur in approximately 50%–60% of ECD. Although the frequency of RDD is lower than that of LCH, JXG, and ECD, it is associated with genetic mutations in the MAPK/ERK pathway, such as KRAS, NRAS, MAP2K1, ARAF, CSF1R, and, only rarely, BRAF V600E. By comprehensively evaluating these characteristics, LCH can be distinguished from other histiocytic diseases [2]. The primary immunohistochemical markers of LCH include S-100 protein, CD1a, and CD207, which are consistently expressed in LCH cells. Molecular pathology plays a role in identifying mutations such as BRAF V600E (exon 15), which is useful for diagnosing the disease and understanding its behavior [11]. Molecular pathological examination is recommended when a definitive diagnosis cannot be established using the major immunohistochemical markers for LCH, such as S-100 protein, CD1a, and CD207. In cases where whole-body CT or other imaging studies reveal multiorgan involvement, other histiocytic disorders should be taken into consideration, and molecular pathological examination should be actively performed. There are no established criteria for long-term follow-up; however, since it is necessary to evaluate both local progression and systemic organ involvement, we adopt a policy of performing whole-body CT scans approximately every 6 months. If lesions occur outside the local site, additional molecular pathological examination should be conducted to guide the consideration of effective pharmacological therapies.

Therapeutic strategies for LCH differ depending on the disease type. Therapy for SS-type bone lesions, such as in this case, is currently based on empirical approaches, including surgical resection, local steroid injection, combination chemotherapy, and radiotherapy, but there is no sufficient consensus on a standard regimen [5]. Surgical resection is generally performed for SS- and SM-types, which can be controlled with local therapy. Radiotherapy is used alone or in combination in cases in which surgical resection is challenging, and good results have been reported [12]. In the case of SS- and SM-type LCH that occur in the jawbone, favorable outcomes have been reported using surgical resection in many cases, and surgery is the most recommended treatment [13]. There are no established guidelines for radiation doses in radiotherapy, but LCH is generally considered highly radiosensitive and can be effectively controlled with low-dose irradiation of approximately 20 Gy [11]. In cases refractory to surgery or radiotherapy, systemic chemotherapy is recommended, and typically involving multidrug regimens incorporating chemotherapy such as vincristine and methotrexate is administered [12].

The efficacy of steroids for LCH of the bone was first reported by Cohen et al. in 1980, who administered local injections of methylprednisolone to eight cases of LCH and observed subsequent healing [14]. The mechanism of action of steroids in LCH is thought to involve the modulation of Langerhans cell, T lymphocyte, and eosinophil activity, as well as the promotion of bone formation; however, the precise details remain unclear [14]. Currently, there are no established guidelines regarding the type, dosage, or number of times steroids should be administered. Esen et al. suggested that, in cases involving extensive lesions, a single dose may be insufficient to cover the entire affected area, necessitating multiple doses [16]. They recommended that the drug be administered repeatedly over a period of at least four to 6 weeks. For our patient, we selected DEX for our patient and administered a total of 48 mg (6 mg a week) and obtained good results. For SS- and SM-type of LCH, therapies such as observation, surgical resection, local steroids administration, and radiation therapy are recommended. For MM-type of LCH, therapy is selected based on whether or not there are affected risk organs. If the hematopoietic system or liver is unaffected, systemic chemotherapy using steroids and vinblastine is administered. If there are lesions in the risk organs, the combination of steroids, vinblastine, and other drugs such as etoposide and methotrexate is recommended [6]. This was a case of an SS-type of LCH that developed in the mandible of an elderly male patient and caused mandibular destruction. As the patient's pain progressed, the therapeutic strategy was changed from observation to active therapy. There is a concern that radiotherapy may cause osteonecrosis of the mandible, and the side effects of chemotherapy and systemic administration of steroids, such as immunosuppression, are likely to be unacceptable for the elderly. Therapies for LCH that is localized to the mandible of the elderly include surgical resection of the lesion, low-dose radiation therapy, and local administration of steroids [6]. LCH of the mandible in the elderly is the extremely rare disease that tends to be more difficult to diagnose and more likely to progress than in younger adults. Therapy options are often limited due to the patient's overall health status and age-related factors [17]. In selecting a therapeutic strategy, we opted for surgical resection because we were concerned about the development of osteosclerosis and mandibular fractures due to radiation therapy as well as the possibility of side effects from anticancer drugs. In addition, local steroids injection has been used in combination with surgical resection [14]. To the best of our knowledge, there are no previous reports of surgical resection combined with local steroids injection for the SS-type of LCH; however, our case study demonstrates its effectiveness and safety.

We reported a case of LCH that developed in the mandible of an elderly patient. Given the potential for the disease progression to worsen if left untreated, it is crucial to consider LCH in the differential diagnosis of bone lesions in adults. There are no established guidelines for the treatment of LCH that occurs in the mandible of the elderly. Considering the overall health status of elderly individuals, radiotherapy may cause jaw necrosis, and systemic chemotherapy and steroids administration may lead to adverse effects such as immunosuppression. This report suggests that a combination of maximal surgical resection of the lesion and local corticosteroid administration of steroids may be an effective therapy and well-tolerated treatment option for elderly patients.
